# 5-Bromo-2,4,6-trimethyl-3-(4-methyl­phenyl­sulfin­yl)-1-benzo­furan

**DOI:** 10.1107/S1600536814004310

**Published:** 2014-03-05

**Authors:** Hong Dae Choi, Pil Ja Seo, Uk Lee

**Affiliations:** a Department of Chemistry, Dongeui University, San 24 Kaya-dong, Busanjin-gu, Busan 614-714, Republic of Korea; b Department of Chemistry, Pukyong National University, 599-1 Daeyeon 3-dong, Nam-gu, Busan 608-737, Republic of Korea

## Abstract

In the title compound, C_18_H_17_BrO_2_S, the dihedral angle between the methyl­phenyl ring and the mean plane of the benzo­furan rung system is 87.0 (2)°. In the crystal, mol­ecules related by inversion are paired into dimers *via* C—H⋯O and C—H⋯π inter­actions. These dimers are further linked by C—H⋯O hydrogen bonds and π–π inter­actions between the benzene and furan rings of neighbouring mol­ecules [centroid–centroid distance = 3.555 (5) Å], resulting in a three-dimensional supra­molecular network.

## Related literature   

For background information and the crystal structures of related compounds, see: Choi *et al.* (2008 ▶, 2011 ▶).
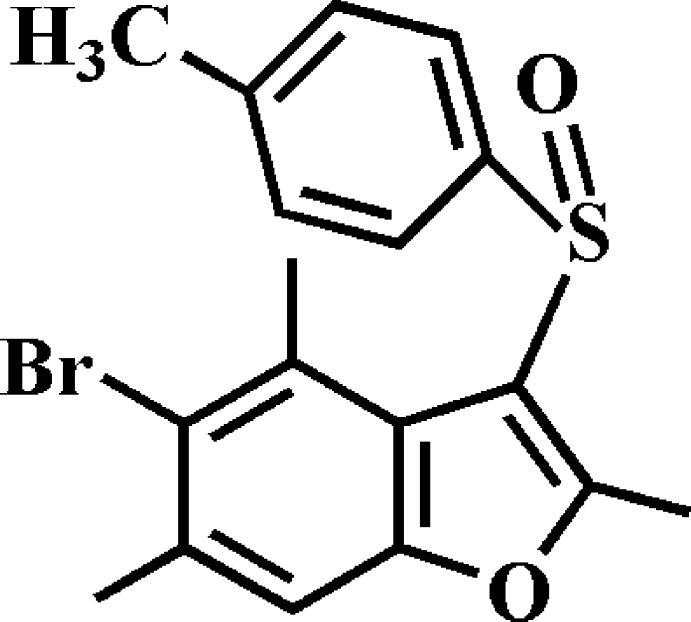


## Experimental   

### 

#### Crystal data  

C_18_H_17_BrO_2_S
*M* *_r_* = 377.29
Triclinic, 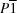

*a* = 8.793 (5) Å
*b* = 9.229 (5) Å
*c* = 10.861 (6) Å
α = 86.105 (16)°
β = 69.582 (17)°
γ = 80.550 (16)°
*V* = 814.7 (8) Å^3^
*Z* = 2
Mo *K*α radiation
μ = 2.66 mm^−1^
*T* = 173 K
0.42 × 0.35 × 0.23 mm

#### Data collection  

Bruker SMART APEXII CCD diffractometer
Absorption correction: multi-scan (*SADABS*; Bruker, 2009 ▶) *T*_min_ = 0.402, *T*_max_ = 0.580
10322 measured reflections
2857 independent reflections
2420 reflections with *I* > 2σ(*I*)
*R*_int_ = 0.067

#### Refinement  

*R*[*F* ^2^ > 2σ(*F* ^2^)] = 0.062
*wR*(*F* ^2^) = 0.177
*S* = 1.15
2857 reflections
203 parameters
H-atom parameters constrained
Δρ_max_ = 1.44 e Å^−3^
Δρ_min_ = −1.27 e Å^−3^

### 

Data collection: *APEX2* (Bruker, 2009 ▶); cell refinement: *SAINT* (Bruker, 2009 ▶); data reduction: *SAINT*; program(s) used to solve structure: *SHELXS97* (Sheldrick, 2008 ▶); program(s) used to refine structure: *SHELXL97* (Sheldrick, 2008 ▶); molecular graphics: *ORTEP-3* for Windows (Farrugia, 2012 ▶) and *DIAMOND* (Brandenburg, 1998 ▶); software used to prepare material for publication: *SHELXL97*.

## Supplementary Material

Crystal structure: contains datablock(s) global, I. DOI: 10.1107/S1600536814004310/xu5773sup1.cif

Structure factors: contains datablock(s) I. DOI: 10.1107/S1600536814004310/xu5773Isup2.hkl

Supporting information file. DOI: 10.1107/S1600536814004310/xu5773Isup3.cml

CCDC reference: 988674

Additional supporting information:  crystallographic information; 3D view; checkCIF report

## Figures and Tables

**Table 1:** Hydrogen-bond geometry (Å, °) *Cg*2 is the centroid of the C1/C2/C7/O1/C8 furan ring.

*D*—H⋯*A*	*D*—H	H⋯*A*	*D*⋯*A*	*D*—H⋯*A*
C6—H6⋯O2^i^	0.95	2.32	3.181 (6)	151
C17—H17⋯O1^ii^	0.95	2.58	3.456 (6)	154
C11—H11*A*⋯*Cg*2^ii^	0.98	2.83	3.794 (6)	167

Symmetry codes: (i) 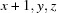; (ii) 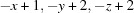.

**Fig. 1.**
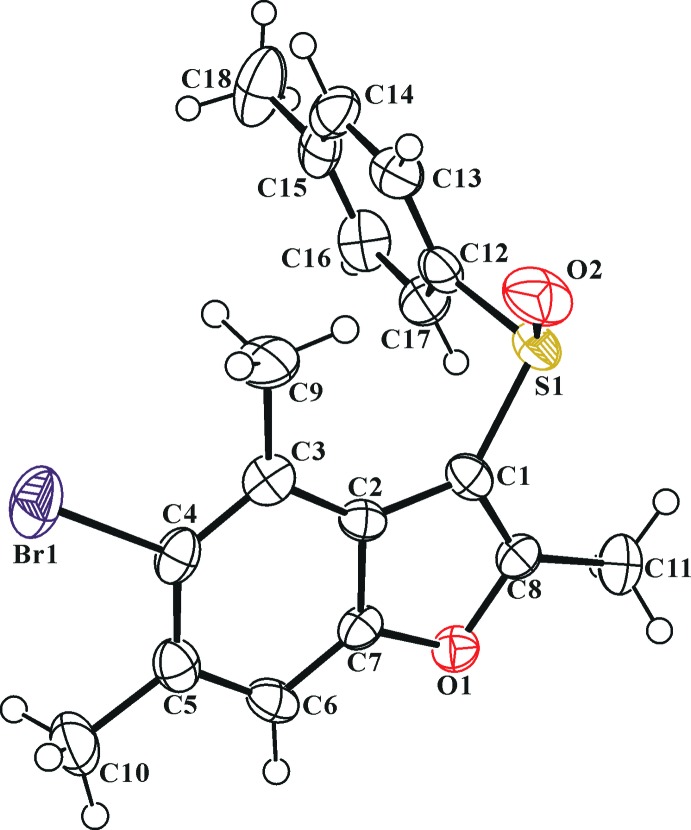
The molecular structure of the title compound with the atom numbering scheme. Displacement ellipsoids are drawn at the 50% probability level. H atoms are presented as small spheres of arbitrary radius.

**Fig. 2.**
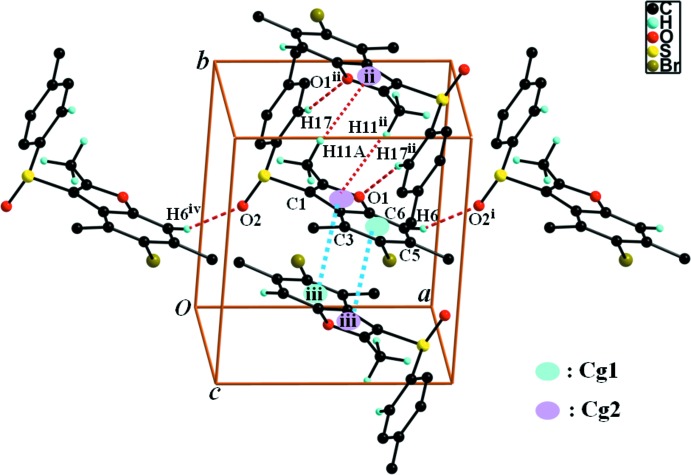
A view of the C—H···O, C—H···π and π···π interactions (dotted lines) in the crystal structure of the title compound. H atoms non-participating in hydrogen-bonding were omitted for clarity. [Symmetry codes: (i) x + 1, y, z; (ii) - x + 1, - y + 2, - z + 2; (iii) - x + 1, - y + 1, - z + 2; (iv) x - 1, y, z.]

## supplementary crystallographic information

### 1. Comment

As a part of our continuing study of 5-bromo-2,4,6-trimethyl-1-benzofuran derivatives containing phenylsulfinyl (Choi *et al.*, 2008) and 4-fluorophenylsulfinyl (Choi *et al.*, 2011) substituents in 3-position, we report here the crystal structure of the title compound.

In the title molecule (Fig. 1), the benzofuran unit is essentially planar, with a mean deviation of 0.017 (3) Å from the least-squares plane defined by the nine constituent atoms. The 4-methylphenyl ring is essentially planar, with a mean deviation of 0.007 (4) Å from the least-squares plane defined by the six constituent atoms. The dihedral angle formed by the benzofuran ring system and the 4-methylphenyl ring is 87.0 (2)°. In the crystal structure, Fig. 2, the molecules related by inversion are paired into dimers via C—H···O and C—H···π interactions (Table 1, Cg2 is the centroid of the C1/C2/C7/O1/C8 furan ring). These dimers are further packed by intermolecular C—H···O hydrogen bonds (Table 1) and π···π interactions between the benzene and furan rings of neighbouring molecules, with a Cg1···Cg2^iii^ distance of 3.555 (5) Å and an interplanar distance of 3.499 (5) Å resulting in a slippage of 0.629 (5) Å (Cg1 is the centroid of the C2–C7 benzene ring), resulting in a three-dimensional network.

### 2. Experimental

3-Chloroperoxybenzoic acid (77%, 224 mg, 1.0 mmol) was added in small portions to a stirred solution of 5-bromo-2,4,6-trimethyl-3-(4-methylphenylsulfanyl)-1-benzofuran (325 mg, 0.9 mmol) in dichloromethane (30 mL) at 273 K. After being stirred at room temperature for 5h, the mixture was washed with saturated sodium bicarbonate solution and the organic layer was separated, dried over magnesium sulfate, filtered and concentrated at reduced pressure. The residue was purified by column chromatography (hexane–ethyl acetate, 2:1 *v*/*v*) to afford the title compound as a colorless solid [yield 69%, m.p. 470–471 K; *R*_f_ = 0.51 (hexane–ethyl acetate, 2:1 *v*/*v*)]. Single crystals suitable for X-ray diffraction were prepared by slow evaporation of a solution of the title compound in ethyl acetate at room temperature.

### 3. Refinement

All H atoms were positioned geometrically and refined using a riding model, with C—H = 0.95 Å for aryl and 0.98 Å for methyl H atoms. *U*_iso_(H) = 1.2*U*_eq_(C) for aryl and 1.5*U*_eq_(C) for methyl H atoms. The positions of methyl hydrogens were optimized rotationally. The highest peak in the difference map is 0.88 Å from BR1 and the deepest hole is 0.95 Å from BR1.

### Crystal data

**Table:**

C_18_H_17_BrO_2_S	*Z* = 2
*M**_r_* = 377.29	*F*(000) = 384
Triclinic, *P*1	*D*_x_ = 1.538 Mg m^−^^3^
Hall symbol: -P 1	Melting point = 470–471 K
*a* = 8.793 (5) Å	Mo *K*α radiation, λ = 0.71073 Å
*b* = 9.229 (5) Å	Cell parameters from 5866 reflections
*c* = 10.861 (6) Å	θ = 2.2–28.2°
α = 86.105 (16)°	µ = 2.66 mm^−^^1^
β = 69.582 (17)°	*T* = 173 K
γ = 80.550 (16)°	Block, colourless
*V* = 814.7 (8) Å^3^	0.42 × 0.35 × 0.23 mm

### Data collection

**Table:**

Bruker SMART APEXII CCD diffractometer	2857 independent reflections
Radiation source: rotating anode	2420 reflections with *I* > 2σ(*I*)
Graphite multilayer monochromator	*R*_int_ = 0.067
Detector resolution: 10.0 pixels mm^-1^	θ_max_ = 25.0°, θ_min_ = 2.0°
φ and ω scans	*h* = −10→10
Absorption correction: multi-scan (*SADABS*; Bruker, 2009)	*k* = −10→10
*T*_min_ = 0.402, *T*_max_ = 0.580	*l* = −12→12
10322 measured reflections	

### Refinement

**Table:**

Refinement on *F*^2^	Primary atom site location: structure-invariant direct methods
Least-squares matrix: full	Secondary atom site location: difference Fourier map
*R*[*F*^2^ > 2σ(*F*^2^)] = 0.062	Hydrogen site location: difference Fourier map
*wR*(*F*^2^) = 0.177	H-atom parameters constrained
*S* = 1.15	*w* = 1/[σ^2^(*F*_o_^2^) + (0.0949*P*)^2^ + 1.1374*P*] where *P* = (*F*_o_^2^ + 2*F*_c_^2^)/3
2857 reflections	(Δ/σ)_max_ = 0.001
203 parameters	Δρ_max_ = 1.44 e Å^−^^3^
0 restraints	Δρ_min_ = −1.27 e Å^−^^3^

### Special details

**Table:**

Geometry. All esds (except the esd in the dihedral angle between two l.s. planes) are estimated using the full covariance matrix. The cell esds are taken into account individually in the estimation of esds in distances, angles and torsion angles; correlations between esds in cell parameters are only used when they are defined by crystal symmetry. An approximate (isotropic) treatment of cell esds is used for estimating esds involving l.s. planes.
Refinement. Refinement of F^2^ against ALL reflections. The weighted R-factor wR and goodness of fit S are based on F^2^, conventional R-factors R are based on F, with F set to zero for negative F^2^. The threshold expression of F^2^ > 2sigma(F^2^) is used only for calculating R-factors(gt) etc. and is not relevant to the choice of reflections for refinement. R-factors based on F^2^ are statistically about twice as large as those based on F, and R- factors based on ALL data will be even larger.

### Fractional atomic coordinates and isotropic or equivalent isotropic displacement parameters (Å^2^)

**Table:**

	*x*	*y*	*z*	*U*_iso_*/*U*_eq_	
Br1	0.73930 (7)	0.37481 (6)	0.59473 (5)	0.0546 (3)	
S1	0.14037 (13)	0.83306 (15)	0.96038 (13)	0.0394 (3)	
O1	0.5385 (4)	0.7754 (3)	1.0502 (3)	0.0328 (7)	
O2	0.0597 (5)	0.7075 (5)	0.9495 (4)	0.0571 (11)	
C1	0.3381 (5)	0.7704 (5)	0.9688 (5)	0.0319 (10)	
C2	0.4792 (5)	0.6678 (5)	0.8932 (4)	0.0276 (9)	
C3	0.5165 (6)	0.5721 (5)	0.7880 (5)	0.0346 (10)	
C4	0.6773 (6)	0.4989 (5)	0.7443 (5)	0.0346 (10)	
C5	0.7984 (6)	0.5111 (5)	0.7983 (5)	0.0360 (11)	
C6	0.7561 (5)	0.6025 (5)	0.9048 (5)	0.0346 (11)	
H6	0.8326	0.6129	0.9463	0.042*	
C7	0.5984 (5)	0.6779 (5)	0.9481 (4)	0.0278 (9)	
C8	0.3796 (5)	0.8293 (5)	1.0595 (4)	0.0307 (10)	
C9	0.3914 (7)	0.5491 (7)	0.7294 (6)	0.0503 (14)	
H9A	0.4179	0.5943	0.6416	0.075*	
H9B	0.2824	0.5945	0.7855	0.075*	
H9C	0.3921	0.4436	0.7229	0.075*	
C10	0.9707 (6)	0.4268 (7)	0.7463 (6)	0.0520 (14)	
H10A	1.0319	0.4439	0.8028	0.078*	
H10B	1.0265	0.4603	0.6566	0.078*	
H10C	0.9653	0.3216	0.7459	0.078*	
C11	0.2953 (7)	0.9413 (6)	1.1636 (5)	0.0447 (12)	
H11A	0.3449	1.0314	1.1384	0.067*	
H11B	0.3071	0.9033	1.2468	0.067*	
H11C	0.1786	0.9629	1.1742	0.067*	
C12	0.1975 (5)	0.9215 (5)	0.8030 (5)	0.0353 (11)	
C13	0.1410 (6)	0.8845 (6)	0.7076 (6)	0.0413 (12)	
H13	0.0813	0.8043	0.7215	0.050*	
C14	0.1720 (7)	0.9653 (6)	0.5911 (5)	0.0450 (12)	
H14	0.1340	0.9392	0.5250	0.054*	
C15	0.2570 (7)	1.0826 (6)	0.5702 (5)	0.0459 (13)	
C16	0.3127 (7)	1.1186 (6)	0.6677 (6)	0.0485 (14)	
H16	0.3734	1.1980	0.6538	0.058*	
C17	0.2810 (6)	1.0405 (6)	0.7841 (5)	0.0414 (12)	
H17	0.3162	1.0681	0.8513	0.050*	
C18	0.2899 (11)	1.1726 (8)	0.4462 (6)	0.075 (2)	
H18A	0.3956	1.1316	0.3821	0.112*	
H18B	0.2933	1.2741	0.4653	0.112*	
H18C	0.2025	1.1711	0.4101	0.112*	

### Atomic displacement parameters (Å^2^)

**Table:**

	*U*^11^	*U*^22^	*U*^33^	*U*^12^	*U*^13^	*U*^23^
Br1	0.0716 (5)	0.0430 (4)	0.0419 (4)	−0.0002 (3)	−0.0117 (3)	−0.0140 (3)
S1	0.0234 (5)	0.0507 (7)	0.0420 (7)	−0.0043 (5)	−0.0094 (5)	0.0013 (6)
O1	0.0333 (16)	0.0353 (17)	0.0331 (18)	−0.0088 (13)	−0.0132 (13)	−0.0042 (14)
O2	0.0391 (19)	0.068 (3)	0.070 (3)	−0.0237 (18)	−0.0217 (19)	0.015 (2)
C1	0.026 (2)	0.031 (2)	0.037 (3)	−0.0052 (17)	−0.0097 (18)	0.0021 (19)
C2	0.026 (2)	0.028 (2)	0.033 (2)	−0.0085 (16)	−0.0128 (17)	0.0018 (18)
C3	0.042 (3)	0.029 (2)	0.036 (3)	−0.0090 (19)	−0.015 (2)	0.001 (2)
C4	0.046 (3)	0.028 (2)	0.028 (2)	−0.0071 (19)	−0.0085 (19)	−0.0049 (18)
C5	0.033 (2)	0.034 (2)	0.036 (3)	−0.0050 (19)	−0.0055 (19)	0.003 (2)
C6	0.024 (2)	0.039 (3)	0.044 (3)	−0.0084 (18)	−0.014 (2)	0.005 (2)
C7	0.031 (2)	0.031 (2)	0.026 (2)	−0.0122 (17)	−0.0111 (17)	−0.0006 (17)
C8	0.034 (2)	0.031 (2)	0.027 (2)	−0.0066 (18)	−0.0106 (18)	0.0022 (18)
C9	0.051 (3)	0.053 (3)	0.059 (4)	−0.006 (2)	−0.032 (3)	−0.018 (3)
C10	0.035 (3)	0.059 (3)	0.048 (3)	0.002 (2)	−0.001 (2)	−0.002 (3)
C11	0.046 (3)	0.044 (3)	0.037 (3)	−0.005 (2)	−0.003 (2)	−0.009 (2)
C12	0.027 (2)	0.034 (2)	0.041 (3)	0.0061 (18)	−0.0106 (19)	−0.005 (2)
C13	0.039 (3)	0.037 (3)	0.050 (3)	−0.004 (2)	−0.018 (2)	−0.006 (2)
C14	0.057 (3)	0.041 (3)	0.037 (3)	0.006 (2)	−0.020 (2)	−0.010 (2)
C15	0.056 (3)	0.032 (3)	0.038 (3)	0.006 (2)	−0.007 (2)	−0.010 (2)
C16	0.053 (3)	0.034 (3)	0.054 (4)	−0.009 (2)	−0.010 (3)	−0.001 (2)
C17	0.044 (3)	0.040 (3)	0.043 (3)	−0.008 (2)	−0.017 (2)	−0.006 (2)
C18	0.111 (6)	0.051 (4)	0.040 (4)	0.001 (4)	−0.006 (4)	0.002 (3)

### Geometric parameters (Å, º)

**Table:**

Br1—C4	1.918 (5)	C10—H10A	0.9800
S1—O2	1.485 (4)	C10—H10B	0.9800
S1—C1	1.772 (5)	C10—H10C	0.9800
S1—C12	1.785 (5)	C11—H11A	0.9800
O1—C8	1.374 (6)	C11—H11B	0.9800
O1—C7	1.375 (6)	C11—H11C	0.9800
C1—C8	1.337 (7)	C12—C13	1.378 (8)
C1—C2	1.456 (6)	C12—C17	1.384 (7)
C2—C7	1.392 (6)	C13—C14	1.389 (8)
C2—C3	1.405 (7)	C13—H13	0.9500
C3—C4	1.393 (7)	C14—C15	1.378 (8)
C3—C9	1.497 (7)	C14—H14	0.9500
C4—C5	1.406 (7)	C15—C16	1.391 (9)
C5—C6	1.384 (7)	C15—C18	1.497 (8)
C5—C10	1.515 (7)	C16—C17	1.375 (8)
C6—C7	1.378 (6)	C16—H16	0.9500
C6—H6	0.9500	C17—H17	0.9500
C8—C11	1.490 (7)	C18—H18A	0.9800
C9—H9A	0.9800	C18—H18B	0.9800
C9—H9B	0.9800	C18—H18C	0.9800
C9—H9C	0.9800		
			
O2—S1—C1	110.8 (2)	C5—C10—H10B	109.5
O2—S1—C12	106.7 (2)	H10A—C10—H10B	109.5
C1—S1—C12	99.4 (2)	C5—C10—H10C	109.5
C8—O1—C7	106.3 (3)	H10A—C10—H10C	109.5
C8—C1—C2	107.6 (4)	H10B—C10—H10C	109.5
C8—C1—S1	117.8 (4)	C8—C11—H11A	109.5
C2—C1—S1	134.5 (4)	C8—C11—H11B	109.5
C7—C2—C3	119.4 (4)	H11A—C11—H11B	109.5
C7—C2—C1	104.1 (4)	C8—C11—H11C	109.5
C3—C2—C1	136.6 (4)	H11A—C11—H11C	109.5
C4—C3—C2	114.9 (4)	H11B—C11—H11C	109.5
C4—C3—C9	122.9 (5)	C13—C12—C17	120.2 (5)
C2—C3—C9	122.2 (4)	C13—C12—S1	120.4 (4)
C3—C4—C5	125.7 (4)	C17—C12—S1	118.8 (4)
C3—C4—Br1	116.9 (4)	C12—C13—C14	119.5 (5)
C5—C4—Br1	117.4 (4)	C12—C13—H13	120.2
C6—C5—C4	118.0 (4)	C14—C13—H13	120.2
C6—C5—C10	119.3 (5)	C15—C14—C13	120.9 (5)
C4—C5—C10	122.7 (5)	C15—C14—H14	119.6
C7—C6—C5	117.2 (4)	C13—C14—H14	119.6
C7—C6—H6	121.4	C14—C15—C16	118.8 (5)
C5—C6—H6	121.4	C14—C15—C18	121.9 (6)
O1—C7—C6	124.4 (4)	C16—C15—C18	119.3 (6)
O1—C7—C2	110.8 (4)	C17—C16—C15	120.8 (5)
C6—C7—C2	124.8 (4)	C17—C16—H16	119.6
C1—C8—O1	111.2 (4)	C15—C16—H16	119.6
C1—C8—C11	134.7 (5)	C16—C17—C12	119.8 (5)
O1—C8—C11	114.0 (4)	C16—C17—H17	120.1
C3—C9—H9A	109.5	C12—C17—H17	120.1
C3—C9—H9B	109.5	C15—C18—H18A	109.5
H9A—C9—H9B	109.5	C15—C18—H18B	109.5
C3—C9—H9C	109.5	H18A—C18—H18B	109.5
H9A—C9—H9C	109.5	C15—C18—H18C	109.5
H9B—C9—H9C	109.5	H18A—C18—H18C	109.5
C5—C10—H10A	109.5	H18B—C18—H18C	109.5
			
O2—S1—C1—C8	−134.4 (4)	C5—C6—C7—C2	0.5 (7)
C12—S1—C1—C8	113.6 (4)	C3—C2—C7—O1	−179.3 (4)
O2—S1—C1—C2	48.7 (5)	C1—C2—C7—O1	1.0 (5)
C12—S1—C1—C2	−63.3 (5)	C3—C2—C7—C6	1.7 (7)
C8—C1—C2—C7	−0.9 (5)	C1—C2—C7—C6	−178.0 (4)
S1—C1—C2—C7	176.2 (4)	C2—C1—C8—O1	0.6 (5)
C8—C1—C2—C3	179.5 (5)	S1—C1—C8—O1	−177.1 (3)
S1—C1—C2—C3	−3.4 (8)	C2—C1—C8—C11	178.4 (5)
C7—C2—C3—C4	−2.6 (6)	S1—C1—C8—C11	0.7 (7)
C1—C2—C3—C4	177.0 (5)	C7—O1—C8—C1	0.0 (5)
C7—C2—C3—C9	176.4 (5)	C7—O1—C8—C11	−178.3 (4)
C1—C2—C3—C9	−4.1 (8)	O2—S1—C12—C13	10.6 (4)
C2—C3—C4—C5	1.6 (7)	C1—S1—C12—C13	125.8 (4)
C9—C3—C4—C5	−177.3 (5)	O2—S1—C12—C17	−177.8 (4)
C2—C3—C4—Br1	−176.7 (3)	C1—S1—C12—C17	−62.6 (4)
C9—C3—C4—Br1	4.4 (6)	C17—C12—C13—C14	1.6 (7)
C3—C4—C5—C6	0.5 (7)	S1—C12—C13—C14	173.0 (4)
Br1—C4—C5—C6	178.8 (3)	C12—C13—C14—C15	−0.6 (8)
C3—C4—C5—C10	179.4 (5)	C13—C14—C15—C16	0.4 (8)
Br1—C4—C5—C10	−2.3 (6)	C13—C14—C15—C18	−179.2 (5)
C4—C5—C6—C7	−1.5 (6)	C14—C15—C16—C17	−1.3 (8)
C10—C5—C6—C7	179.5 (4)	C18—C15—C16—C17	178.4 (5)
C8—O1—C7—C6	178.3 (4)	C15—C16—C17—C12	2.2 (8)
C8—O1—C7—C2	−0.7 (4)	C13—C12—C17—C16	−2.4 (7)
C5—C6—C7—O1	−178.3 (4)	S1—C12—C17—C16	−174.0 (4)

### Hydrogen-bond geometry (Å, º)

Cg2 is the centroid of the C1/C2/C7/O1/C8 furan ring.

**Table:**

*D*—H···*A*	*D*—H	H···*A*	*D*···*A*	*D*—H···*A*
C6—H6···O2^i^	0.95	2.32	3.181 (6)	151
C17—H17···O1^ii^	0.95	2.58	3.456 (6)	154
C11—H11*A*···*Cg*2^ii^	0.98	2.83	3.794 (6)	167

Symmetry codes: (i) *x*+1, *y*, *z*; (ii) −*x*+1, −*y*+2, −*z*+2.
